# Retraction: The Association of Serum Ferritin Levels With Non-scarring Alopecia in Women

**DOI:** 10.7759/cureus.r136

**Published:** 2024-02-06

**Authors:** Muhammad Fawad Aslam, Mariam Khalid, Muhammad Amad Aslam

**Affiliations:** 1 Department of Medicine, Rai Medical College, Sargodha, PAK; 2 Department of Medical Education, Rai Medical College, Sargodha, PAK; 3 Department of Pathology, Rai Medical College, Sargodha, PAK

The Editors-in-Chief have retracted this article due to plagiarism. Concerns were raised regarding data similarities with the following article published by Chisti et al.: https://www.jpad.com.pk/index.php/jpad/article/view/893/858. The journal's review of these similarities revealed identical study design, details and data in both articles. The article was checked for plagiarism via an automated text duplication checking tool prior to publication but these similarities were not identified at that time.

